# Embodied intelligence for drumming; a reinforcement learning approach to drumming robots

**DOI:** 10.3389/frobt.2024.1450097

**Published:** 2024-11-18

**Authors:** Seyed Mojtaba Karbasi, Alexander Refsum Jensenius, Rolf Inge Godøy, Jim Torresen

**Affiliations:** ^1^ RITMO Centre for Interdisciplinary Studies in Rhythm, Time and Motion, University of Oslo, Oslo, Norway; ^2^ Department of Informatics, University of Oslo, Oslo, Norway; ^3^ Department of Musicology, University of Oslo, Oslo, Norway

**Keywords:** reinforcement learning, embodied intelligence, intrinsic motivation, musical robots, drumming

## Abstract

This paper investigates the potential of the intrinsically motivated reinforcement learning (IMRL) approach for robotic drumming. For this purpose, we implemented an IMRL-based algorithm for a drumming robot called *ZRob*, an underactuated two-DoF robotic arm with flexible grippers. Two ZRob robots were instructed to play rhythmic patterns derived from MIDI files. The RL algorithm is based on the deep deterministic policy gradient (DDPG) method, but instead of relying solely on extrinsic rewards, the robots are trained using a combination of both extrinsic and intrinsic reward signals. The results of the training experiments show that the utilization of intrinsic reward can lead to meaningful novel rhythmic patterns, while using only extrinsic reward would lead to predictable patterns identical to the MIDI inputs. Additionally, the observed drumming patterns are influenced not only by the learning algorithm but also by the robots’ physical dynamics and the drum’s constraints. This work suggests new insights into the potential of embodied intelligence for musical performance.

## 1 Introduction

Music performance is a complex form of skilled sequential action, often with a creative behavioural element. During practice, the musician explores the dynamics of the instrument, their bodily actions, and physical characteristics of the environment to achieve motor skills for playing the instrument (Godøy, 2018). The reference signals that enable the musicians to refine their actions are derived from the musical output of the performance. When the desired musical goal is determined from the beginning, the reference signal is provided by an extrinsic source, e.g., sheet music. Furthermore, exploring the dynamics of performance can lead to novel patterns and creative behaviour, which are not necessarily the primary goal of practice. This is similar to autonomous exploration in children, where they are not told what to learn but instead learn from exploring the environment with curiosity (Oudeyer, 2018; Schmidhuber, 2006). Inspired by these approaches to learning musical performance, we can develop intelligent musical machines, such as robots, that can perform music autonomously and adapt to different situations.

Utilising an extrinsic reference is similar to using ground-truth information in a labelled dataset for supervised learning or a reward signal provided by the environment in reinforcement learning. In this approach, the main assumption is that the desired performance is known. For instance, playing a specific note with a given duration in a specific moment is an example of an extrinsic reference point. The goal of any training process that only uses this type of information is to achieve a result with a minimum difference from the given reference data. Consequently, the resulting performance would be an optimal replication of the known target. On the other hand, intrinsic motivation can add another dimension to the training process in music performance. In machine learning, the intrinsic reward refers to an internal measure based on prediction and surprise factors (Sutton and Barto, 2018). Usually, the reason for using intrinsic reward is to discover novel patterns during the training process. In music performance, the intrinsic reward can lead to finding emergent patterns.

Building on these concepts, the integration of intrinsic motivation into the design of intelligent musical machines presents great potential to explore creativity. Unlike the extrinsic reference approach, where performance targets are pre-defined, and the goal is accurate replication, an intrinsic motivation model would allow these machines to self-direct their learning process. This could resemble a form of artistic exploration, where the machine is not just replicating known pieces but also creating and discovering new musical expressions.

**FIGURE 1:**
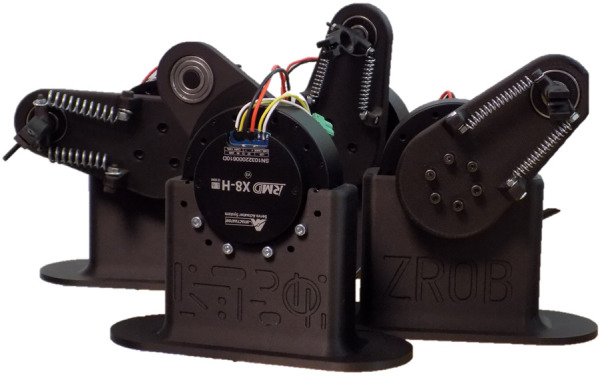
ZRob robots are drumming arms that are actuated by a servo motor and have a flexible gripper. In this photo, four ZRobs are shown. Different springs are used for each arm.

This paper examines the possibility and potential of utilizing intrinsic and extrinsic reward signals in a music performance application. An important aspect of musical robots is the ability to adapt and explore new possibilities (Weinberg et al., 2020). One solution is to deploy different rewarding strategies for training musical robots (Vear, 2021). The training methods have been designed and tested for a physical drumming robot called ZRob (Figure 1). The robot is inspired by the human hand morphology, having a flexible gripper with passive springs. This makes it possible to exploit the rebounding forces in drumming, like in human drumming. Playing multiple-stroke rolls created by the gripper’s vibrations is an example of how embodiment and physical constraints contribute to the drumming performance. Multiple ZRob arms can be combined to generate complex rhythmic patterns. We typically work with pairs of robots, emulating the way a human would play on a drum, such as a snare drum.

Robotic drumming, in general, contains nonlinear and complex dynamics. The physical constraints of the body of ZRob robots also shape the drumming dynamics. The advantage of using an intrinsically motivated learning algorithm for such robots is the possibility of exploring the dynamics and physical constraints of the robot and the drum to discover novel patterns. This approach can also be addressed in the context of *embodied intelligence*, meaning that intelligent behaviour emerges from the interaction between an agent’s brain, body, and environment rather than being a product of computational processes in the brain (Gupta et al., 2021). An essential element of embodied intelligence is the exploitation of the dynamics of the body and the physical constraints of the environment (Roy et al., 2021). In our proposed system, the interaction of the drum and ZRob’s body, especially affected by the flexible gripper, plays a significant role in learning. We have used two robots with different spring stiffness, leading to more diverse emergent drumming patterns. These emergent patterns have been explored in another study without using any learning algorithm (Karbasi et al., 2023).

The learning algorithms used in this study are based on the deep deterministic policy gradient (DDPG) method, which has been successfully implemented in continuous control problems in different applications (Lillicrap et al., 2015; Sajadi et al., 2022). DDPG is a reinforcement learning algorithm based on the actor-critic architecture that trains a policy function for continuous action and state spaces. We used MIDI inputs as the reference set point to define the extrinsic reward function. The intrinsic reward signal is calculated based on a prediction model. During training, the prediction model is updated at each step, and the intrinsic reward is calculated based on the improvement of the model.

An internal model in our system is designed to predict the sound features given the robot’s current state and actions, which allows the robot to understand the relationship between its movements and the produced sound. This approach is directly inspired by the theoretical framework presented in Schmidhuber (2010). While this might seem like a purely predictive function, the role of intrinsic reward lies in driving the system to seek improvements in this prediction. The robot does not simply learn to repeat known patterns but rather is artificially curious to explore the dynamics of its body and environment to discover novel and unpredicted outcomes. This exploration creates opportunities for the emergence of new rhythmic variations and behaviors. The intrinsic reward is calculated based on the surprise factor or error in the prediction model, pushing the robot to adjust its actions in ways that are not dictated by the external reward but are shaped by its ongoing interaction with its physical form and the drum, leading to more creative patterns.

The aim of this paper is to contribute to the development of robotic musicianship for drumming performance by implementing intrinsically motivated reinforcement learning to enable ZRob robotic drummers to demonstrate rhythmic creativity. In particular, we demonstrate how the robots can learn in a real-world setting and examine the role of intrinsic rewards in making emergent drumming patterns. In the next section, we will briefly present the previously related works on drum robots and applications of IMRL for robot learning.

## 2 Background

Musical robotics is a small but emerging field based on interdisciplinary collaborations between several artists, researchers, and engineers. While robotic drumming systems have been widely explored, several other robotic systems have been developed for playing different musical instruments, demonstrating the versatility of robotics in artistic and creative applications. For example, robots have been created to play the marimba, such as Shimon, an interactive improvisation system for a robotic marimba player (Hoffman and Weinberg, 2011). Similarly, piano-playing robots have garnered attention, with works like (Hughes et al., 2018) creating an anthropomorphic soft skeleton hand for piano playing, and Wang et al. (2022) using a data-driven simulation framework for expressive piano performance. These developments highlight the range of musical instruments and robotic platforms contributing to this evolving field.

In robotic drumming, exploring actuation mechanisms is a core interest, and many different actuators have been used for robotic drumming systems. Solenoid-based actuation, transducers and Variable Stiffness Actuators (VSA) have shown exciting results in different studies (Kapur et al., 2007; Kim et al., 2014; Brown and Topel, 2019; Berdahl et al., 2007). While each actuation method can be controlled with different strategies, the drumming dynamics can result in different acoustic features. For instance, VSA motors efficiently perform multiple-stroke drum rolls with desired frequencies (Kim et al., 2014).

The control of drumming robots has been investigated by researchers for different tasks (Murphy et al., 2012; Van Rooyen, 2018; Su et al., 2019). One of the most advanced robotic drumming systems developed in recent years is a robotic prosthesis which is controlled by electromyography (EMG) signals to play drum rolls (Gopinath and Weinberg, 2016; Bretan et al., 2016; Yang et al., 2021). Most of the mentioned works have focused on optimizing drumming tasks by control design or data-driven models. In addition, reinforcement learning is used for controlling drumming robots to optimize single-stroke movements (Okui et al., 2022). Moreover, training an internal model through interaction is also suggested in Wu et al. (2017), Liu et al. (2018) to produce a desired single-stroke drumming sound.

The concept of embodied intelligence, where behaviour emerges from the interaction between a robot’s physical form and its environment, is well-aligned with musical robotics. Recent work in this area includes the study on coordinating upper limbs for piano playing through neuro-musculoskeletal modeling, which similarly emphasizes the robot-environment interaction to achieve complex behaviour (Wang et al., 2023). This connection between physicality and task performance underpins the role of reinforcement learning in robotic drumming.

Intrinsically Motivated Reinforcement Learning (IMRL) has become a powerful tool in the field of robotics, particularly for the tasks that require exploration and creativity (Oudeyer, 2018; Oudeyer et al., 2007; Barto et al., 2004). IMRL essentially works by providing a system with internal rewards for novel or informative actions independent of external goals or tasks. This approach is particularly effective when external rewards are sparse or difficult to define. IMRL has been used in robotics to encourage robots to explore their environments autonomously (Sharma et al., 2019; Sharma et al., 2020). This is crucial for developing robots adapting to new or unpredictable situations. For instance, robots have been trained to navigate a cluttered room or manipulate unfamiliar objects, driven by intrinsic rewards for discovering new strategies or improving their proficiency (Oudeyer et al., 2013). When applied to musical context, IMRL holds the potential for investigating creativity and dynamic interaction, proposing new forms of musical expression and educational tools (Schmidhuber, 2010). The integration of IMRL in musical robotics is still an emerging field, and ongoing research will likely uncover even more innovative applications.

ZRob has been used previously for studying different aspects of robotic drumming (Karbasi et al., 2021a; Karbasi et al., 2022; Karbasi et al., 2021b; Karbasi et al., 2023). In Karbasi et al. (2022) by using one Zrob, the effect of the flexible gripper with passive springs on drum rolls is investigated by changing the control variables of the robot. The study’s results suggest that the stiffness of the gripper limits the timing features of the drum rolls in different ranges of frequency and amplitude of the motion. Furthermore, in Karbasi et al. (2023) using two ZRobs with different stiffness ratios, the dynamical characteristics of the robots are used to explore emergent rhythmic patterns in drum rolls. While the previous studies on ZRob do not contain any learning process, the main contribution of this paper compared to the previous studies is the implementation of the learning algorithm in a real-world setting where the robots are trained to play specific patterns. Overall, the previous studies on ZRob focused on unique features in the body and control for drumming, which are utilized in the learning framework proposed in the current study.

## 3 Materials and methods

### 3.1 ZRob: dynamics and control

**FIGURE 2:**
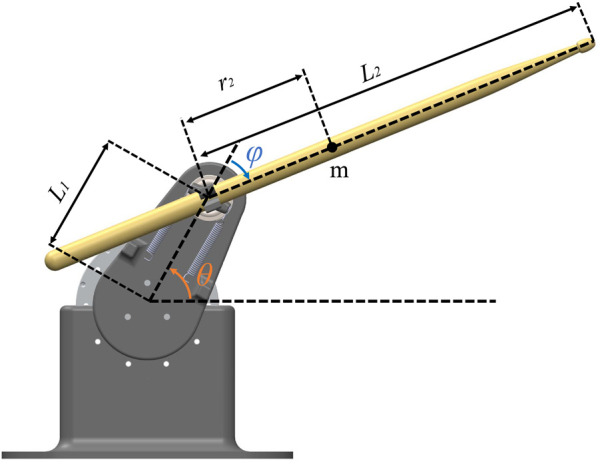
ZRob morphology; an example of Pendubot.

The mechanical design and dynamics of ZRob are described in detail in Karbasi et al. (2023). ZRob is a 2-DoF robotic arm with a flexible joint. ZRob is underactuated and is similar to the Pendubot with passive springs connected to the second joint (Spong et al., 2020). The Pendubot is a double pendulum which is only actuated by one motor in the first joint. In our case, ZRob has an actuator in the first joint and passive springs in the second joint to make the gripper flexible and effective during dynamic interactions with the drum membrane (see Figure 2). The dynamic equations are of the form

$$M\left(\theta ,\varphi \right)\left[\begin{array}{c} \ddot{\theta }\\ \ddot{\varphi } \end{array}\right]+C\left(\theta ,\varphi ,\dot{\theta },\dot{\varphi }\right)\left[\begin{array}{c} \dot{\theta }\\ \dot{\varphi } \end{array}\right]=\tau$$ (1)

In Equation 1 M(θ,φ) is the inertia matrix, $C\left(\theta ,\varphi ,\dot{\theta },\dot{\varphi }\right)$ is the Coriolis matrix and τ is the torque vector.

The actuator of the robot is a quasi-direct drive servo motor with a low transmission ratio (1:6) that enables the controller to precisely adjust the torque according to the angle and velocity set points. It is driven by an internal PID controller with 3 control modes (position, velocity, torque).

In this work, we use the combination of position and velocity control modes of the motor to produce the desired trajectory. The trajectories are generated by an Arduino board communicating with the motors through the CAN-BUS protocol. As a result, we can assume that the angle of the motor θ is an independent controlled variable. Therefore, the first dynamical equation of the robot can be ignored, and the motion of the second joint will follow the dynamic of the form

$$\tau_{2}=\left(\delta +\beta cos\left(\varphi \right)\right)\ddot{\theta }+\delta \ddot{\varphi }+\beta {\dot{\theta }}^{2}$$ (2)

where

$$\delta =I+mr_{2}^{2}$$ (3)

$$\beta =mL_{1}r_{2}$$ (4)

$$\tau_{2}=\tau_{\mathrm{ext}}+\tau_{k}-\tau_{d}-\tau_{\mathrm{grav}}$$ (5)

In Equations 2–5, $\tau_{k}$ is the torque generated by the passive springs, $\tau_{d}$ is the damping torque, $\tau_{\mathrm{grav}}$ is the torque caused by gravity, $\tau_{\mathrm{ext}}$ is the torque caused by interaction between the drum membrane and the drumstick and I is the inertia of the drumstick.

**FIGURE 3:**
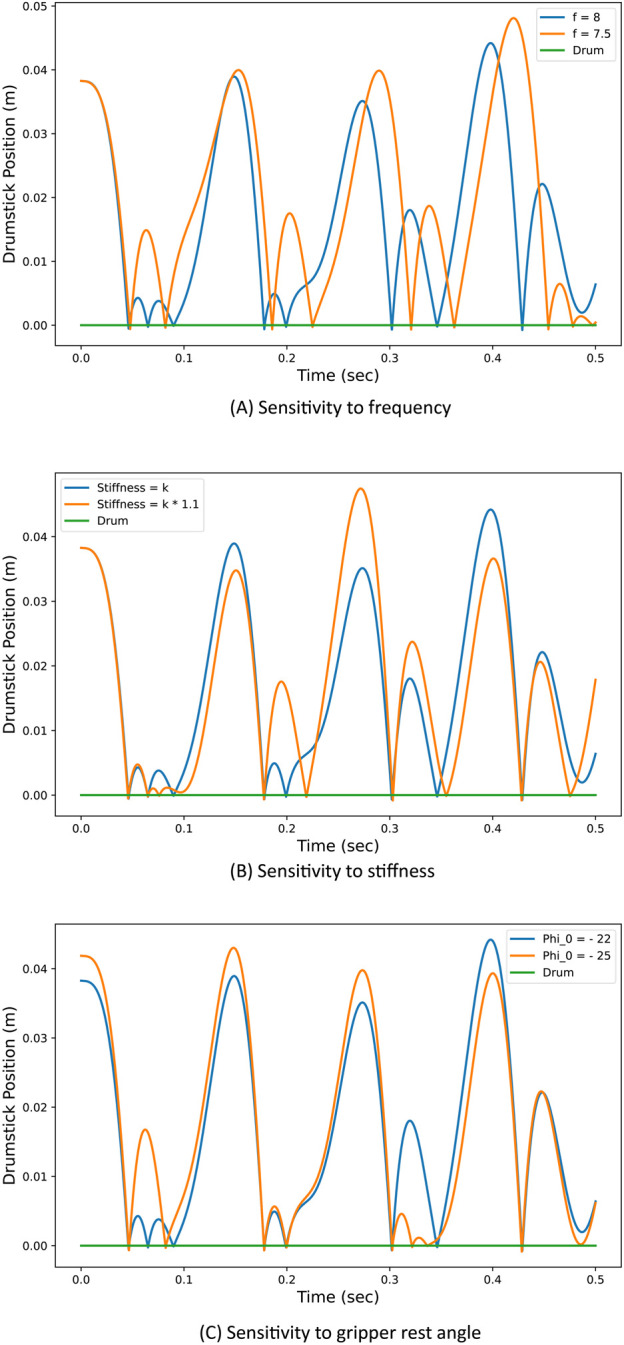
Chaotic response of the drumstick motion with slight parameter changes in simulation. As a result, the drumming patterns alter significantly based on high sensitivity to: **(A)** changes in the frequency of motion, **(B)** changes in spring stiffness, **(C)** or changes in the rest angle of the gripper.

In our previous study (Karbasi et al., 2023), we showed that the resulting dynamic of the robot can lead to chaotic behaviour, and we tried to exploit the natural uncertainty of two ZRobs to create emergent drumming patterns. The chaotic behaviour of the robot can be observed using simulation (Figure 3). The robot’s behaviour is highly sensitive to slight changes in the stiffness of the spring, initial conditions, and positional trajectory of the actuator. The sensitivity analysis of the robots is out of the scope of this study. However, the chaotic nature of the robot’s dynamics is a key element affecting the learning process.

### 3.2 Drumming functions

**FIGURE 4:**
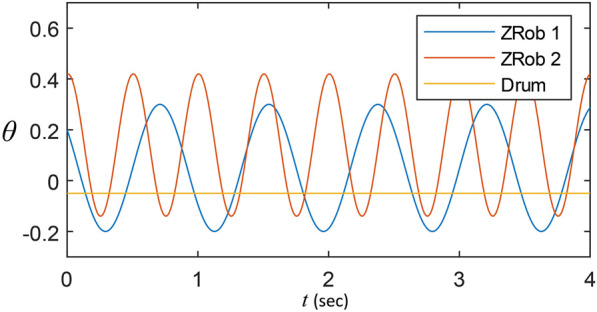
The Trajectories generated by the Arduino to move the robots. Each function is configured by variables such as frequency and amplitude of the movement.

The trajectory generator algorithm uses different functions with adjustable parameters. The functions are sinusoidal trajectory generators sent to the robots to play a drum stroke or roll with desirable parameters. The details of the trajectory generators are described in Karbasi et al. (2023). In Figure 4, an example of a drum roll trajectory with the desired frequency, amplitude, phase shift and vertical shift is illustrated.

Using sinusoidal waveforms enables the robot controller to adjust the motion characteristics such as amplitude and frequency with minimum number of parameters. This is important since increasing the learning parameters would result in a longer duration of training. Additionally, since the robots are underactuated, the motion of the drumstick is mostly affected by the natural response of the flexible joints and using different waveforms can complicate the control of the robot. However, studying the trajectory waveforms can be a topic of further research.

In addition to the drum roll function, which moves two ZRobs simultaneously, each arm is moved separately in another function, generating a single stroke. The single-stroke function has the same trajectory as the drum roll, but it only moves one arm and generates only one periodic cycle of the sinusoidal function. The inputs to the single-stroke function are frequency, amplitude, and vertical shift. In Table 1, all the variables for the functions are described.

**TABLE 1:** ZRob trajectory input variables.

Input	Variable	Range
single-stroke function		
Frequency	$f_{i}$	[1–12] Hz
Amplitude	$A_{i}$	[ π/8 - π/4 ] rad
Vertical shift	$B_{i}$	[0 - π/6 ] rad
roll function		
Frequency	$f_{r}$ , $f_{l}$	[1–15] Hz
Amplitude	$A_{r}$ , $A_{l}$	[ π/8 - π/4 ] rad
Vertical shift	$B_{r}$ , $B_{l}$	[0 - π/6 ] rad
Phase shift	ϕ	[0 - 2π ] rad
Duration	T	[0–30] sec

### 3.3 Learning algorithm

The learning algorithm is based on the DDPG method. As an extrinsic set point, the agent loads an arbitrary MIDI file. The MIDI file is written in a way compatible with the robots and low-level functions executed by the Arduino board.

A MIDI (Musical Instrument Digital Interface) file is a digital format that encodes musical performance data, allowing electronic musical instruments, computers, and other devices to communicate and synchronize with each other. Unlike audio files, which store actual sound recordings, MIDI files contain information such as notes, timing, velocity, and control signals, which are used to trigger the sounds on compatible instruments or software synthesizers. This format is highly versatile, enabling musicians to edit, manipulate, and exchange musical compositions easily without loss of quality, as the data represents the performance rather than the audio itself. For drumming, MIDI files can be used to provide precise instructions for each drum hit, including its timing, duration, and intensity, facilitating accurate and dynamic drumming performances.

We employed the DDPG algorithm, a model-free, off-policy actor-critic method based on deterministic policy gradient that can operate over continuous action spaces. Introduced by Lillicrap et al. (2015), DDPG is an extension of the earlier Deterministic Policy Gradient (DPG) algorithms, incorporating deep neural networks for function approximation. DDPG utilizes two primary networks: the actor and the critic. The actor network is responsible for mapping states to a continuous action space, whereas the critic network estimates the Q-value of the current state and action derived from the actor network.

A key feature of DDPG is the use of target networks for both the actor and the critic, which are slowly updated to stabilize training. This stabilization is further aided by the employment of a replay buffer, a finite-sized cache that stores transitions experienced by the agent, allowing for the reuse of this data across multiple updates. The learning process involves sampling a mini-batch from the replay buffer to update the critic by minimizing the mean squared error between the predicted Q-values and the target Q-values. Subsequently, the actor is updated using the sampled policy gradient. Importantly, DDPG also incorporates noise processes (e.g., Ornstein-Uhlenbeck process) to the action output, promoting exploration in the action space (Lillicrap et al., 2015).

This algorithm has demonstrated effectiveness in various tasks requiring complex, continuous action decisions, making it suitable for our study’s objectives to optimize agent performance in a simulated environment with high-dimensional state and action spaces.

**FIGURE 5:**
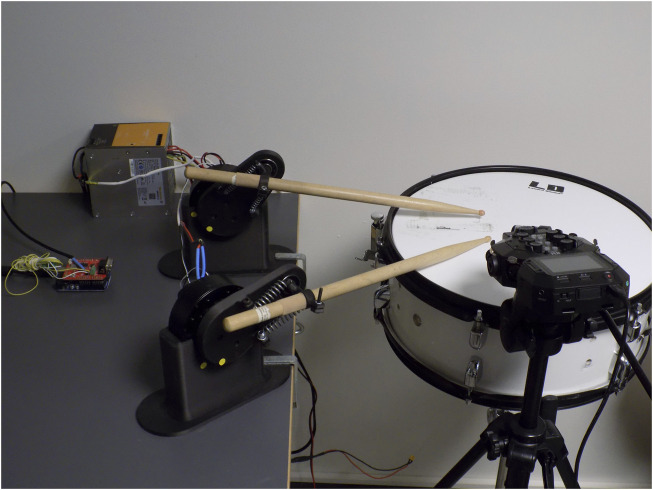
The experimental setup for training the robots.

**FIGURE 6:**
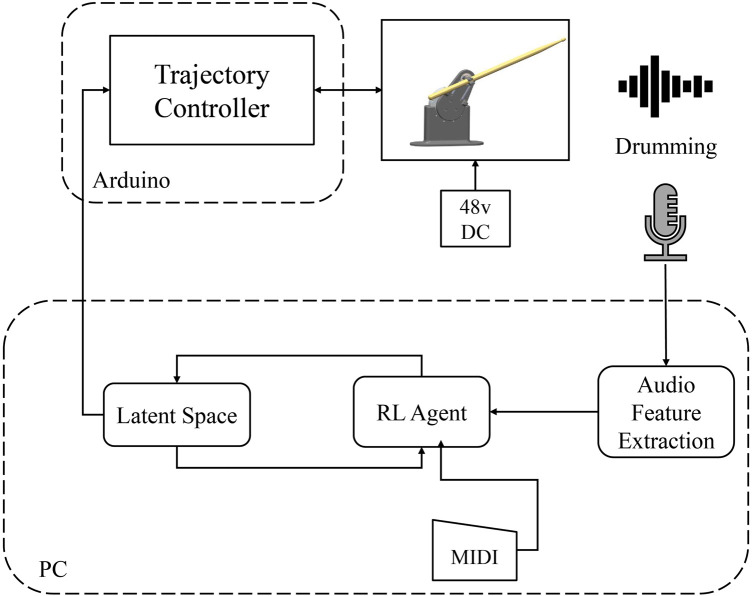
The Reinforcement Learning agent and trajectory controller implementation for the training experiments.

At each step during training, the robots play the notes according to the given MIDI file. The objective of the RL algorithm is to find the best values of the variables defined in Table 1. To train the agent, we have defined a latent space environment. Each point in the latent space corresponds to specific values of the trajectory variables. Once the RL agent chooses a point in the latent space, the trajectory generator executes the drumming functions with the given variables. The resulting drumming sound is recorded using a microphone and processed for calculating the reward. The schematic of the system is shown in Figures 5, 6.

#### 3.3.1 Audio processing and extrinsic reward

The extrinsic reward is defined by comparing the extracted features form the recorded audio and the given MIDI file. The MIDI file specifies onset time and velocity, which can be compared to the extracted onset timings and strength from the recorded audio.

**FIGURE 8:**
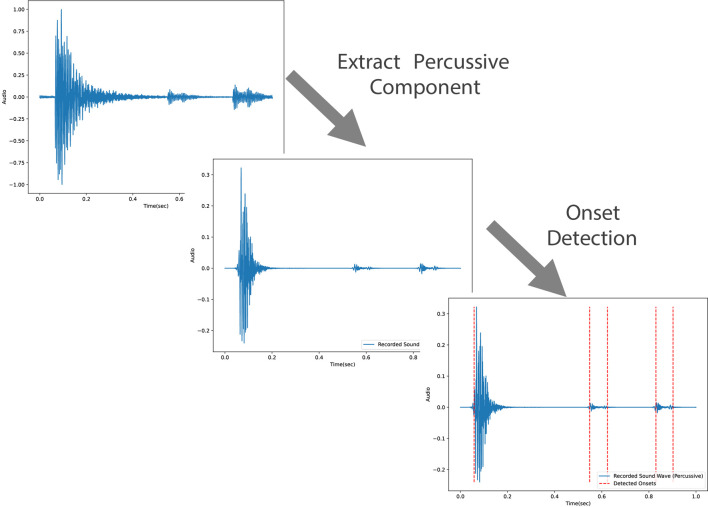
The audio processing unit first extracts the percussive component of the recorded audio. Afterwards, it detects the onsets based on the onset envelope.

In order to detect the onsets in each audio sample, the percussive component of the audio is extracted to remove unwanted elements such as pitch and timbre from the audio. This would improve the accuracy of the onset detection. Using the percussive component of the audio sample, the onsets are extracted by detecting the local peaks of the onset envelope. All of the audio processing steps are implemented using the Librosa library in Python (McFee et al., 2024). The onset detection process is shown in Figure 8.

**FIGURE 9:**
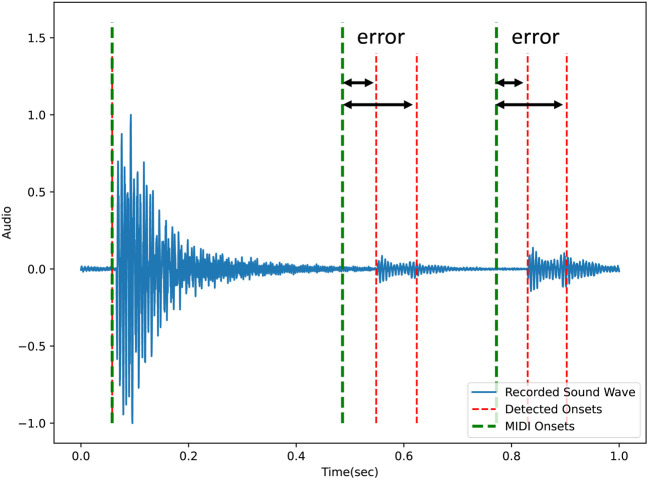
The error between the detected onsets and MIDI onsets is used for calculating the extrinsic reward. The green lines indicate the onsets from the MIDI input and the red lines indicate the detected onsets.

When the onsets are detected, the error between each onset and the reference points given by the MIDI is calculated. In Figure 9, the errors between the detected onsets (red lines) and the MIDI onsets (Green lines) are illustrated. The extrinsic reward is the sum of the calculated errors:

$$r_{\mathrm{ext}}=-\underset{n}{\sum }{\left(t_{\mathrm{ons}}-t_{\mathrm{mid}}\right)}^{2}$$ (6)

In Equation 6 n is the number of onsets, $t_{\mathrm{ons}}$ is the detected onset (red lines), and $t_{\mathrm{mid}}$ is the closest reference point to each onset (green lines) Using only the extrinsic reward, the algorithm learns to minimize the error between the played sound and the given MIDI. The intrinsic reward is calculated based on a surprise factor that indicates the improvement of an internal model during training. A combination of intrinsic and extrinsic rewards is given to the algorithm to update the agent. Algorithm 1 provides the details of the training process of our system.

Algorithm 1 Reinforcement Learning with Intrinsic Motivation.

Initialize *actor*, *critic*, p ;
**while** Episode≤N **do**
 Reset memory
 **while** t≤T **do**
  Take an action a←π(s)

  Drum and record the sound
  Extract onsets from audio
  Update p using new observation $s^{\prime }$

  Store all actions and observations in memory
  Calculate intrinsicreward

  
$r\leftarrow r_{\mathrm{int}}+r_{\mathrm{ext}}$

  Update *actor* and *critic* networks using DDPG
 end while
 Next Episode
**end while**

### 3.4 Internal model and intrinsic reward

**FIGURE 7:**
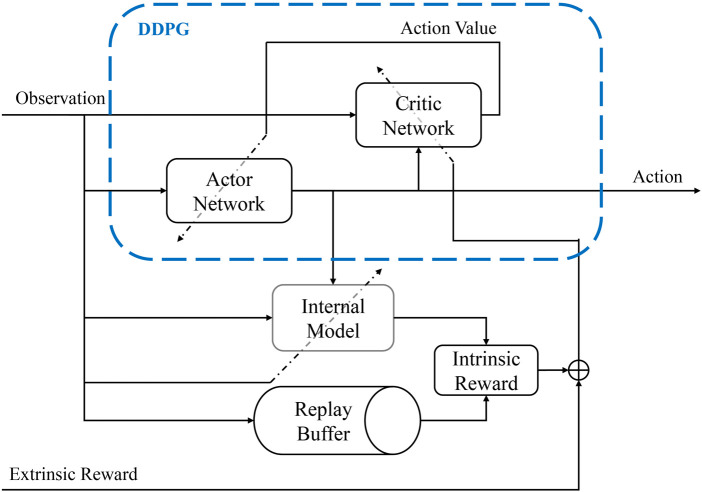
The DDPG algorithm architecture, and the internal model used for calculating the intrinsic reward based on the improvement of the predictions.

To calculate the intrinsic reward for training, the agent needs to use a prediction-based measure. For this purpose, we use an internal model that predicts the sound features of the recorded sound given its current state and action. The intrinsic reward is a surprise factor calculated based on the improvement of the internal model with new observations. The idea of using an internal model for calculating the surprise factor and the intrinsic reward is based on Schmidhuber (2010). Using the intrinsic reward makes the agent explore new patterns in the environment and improve its prediction of the results of the dynamics of its body. Figure 7 depicts how the intrinsic reward is calculated based on the internal model.

The surprise factor is calculated at each time step based on how much the internal model is improved. The internal model is continuously updated with each new observation. In each episode, the precision of the internal model is evaluated based on the stored actions and observations at each time step before and after being updated. Then, the intrinsic reward is the difference between the precision of the internal model before and after taking each action.

Algorithm 2 describes how the intrinsic reward is calculated given the memory buffer and the prediction model. In order to calculate the precision of the internal model, we have used the error function according to Equation 7:

$$E\left(p\right)=\underset{t}{\sum }{\left(p\left(s_{t},a_{t}\right)-s_{t+1}\right)}^{2}$$ (7)

The internal model is a neural network which uses the same input as the critic network in the DDPG algorithm. However, the output of the internal model is the predicted onsets while the critic network predicts the Q-values. In other words, the internal model predicts the onsets that will be played by the robots given the action taken by the actor. During the training, the internal model is updated using the last observation. Since the surprise factor indicates the improvement of the internal model, it should be updated only using the last action and the observed audio to represent the intrinsic reward for the last action. If an action results in a better internal model, the intrinsic reward will be positive.

We have used the same architecture for both internal model and critic networks. The details of the hyper-parameters and implementation of the learning algorithm are described in the next section.

Algorithm 2 Calculating Intrinsic Reward.

**Require:** Memory, p , $p^{\prime }$

 Predict all the observations in memory using p

 Repeat the prediction with the updated model $p^{\prime }$

 
$r_{\mathrm{int}}\leftarrow \left(E\left(p^{\prime }\right)-E\left(p\right)\right)$

## 4 Result

### 4.1 Training in real-world setup

**FIGURE 10:**
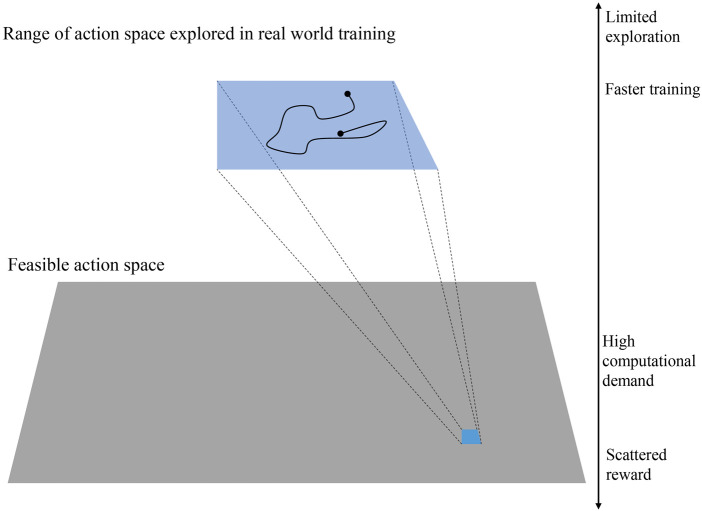
In real-world training, the exploration space should be limited in order to decrease the number of required samples for training and the duration of the training.

**FIGURE 11:**
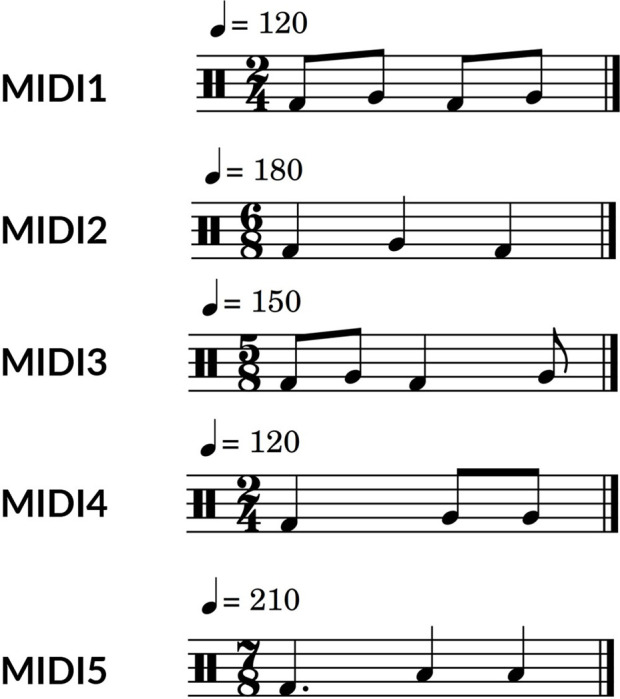
The MIDI inputs used for training.

A big challenge in training a robotic system using RL in the real world is the computation limitation and feasibility. Usually, a large number of samples is needed to get optimal results using RL. In this work, we have limited the range of exploration in the latent space to achieve a meaningful result in a reasonable time. Figure 10 shows the relative size of the exploration range in real-world training. Also, the MIDI inputs we used have a short length (1 s) to make each step short. Having a 1-s MIDI input means that 1 s of drumming is recorded in each training step. For comparison, we trained the robots using 5 different MIDI inputs using only extrinsic reward and a combination of intrinsic and extrinsic reward. The MIDI files are designed in a way to represent different time signatures and rhythmic patterns. The MIDI inputs are given in Figure 11. In training, we used two ZRobs and defined the frequency and amplitude of motion of each ZRob for playing each note as the variables in the latent space. Therefore, the latent space has 4 dimensions, and the number of actions for the agent is also 4.

The models we used for the DDPG and the internal model are feedforward neural networks. The actor-network consists of two layers having 150 and 100 neurons in each layer, respectively. The critic network uses a two-layer input channel for the state with 20 neurons for each layer and a one-layer input channel with 20 neurons for the action. The internal model uses the same architecture as the critic network. The hyperparameters used for training are given in Table 2.

**TABLE 2:** Hyperparameters.

DDPG parameters	
Actor learning rate	0.001
Critic learning rate	0.002
Internal model learning rate	0.0001
τ	0.005
γ	0.99
σ	0.05

### 4.2 Training results

**FIGURE 12:**
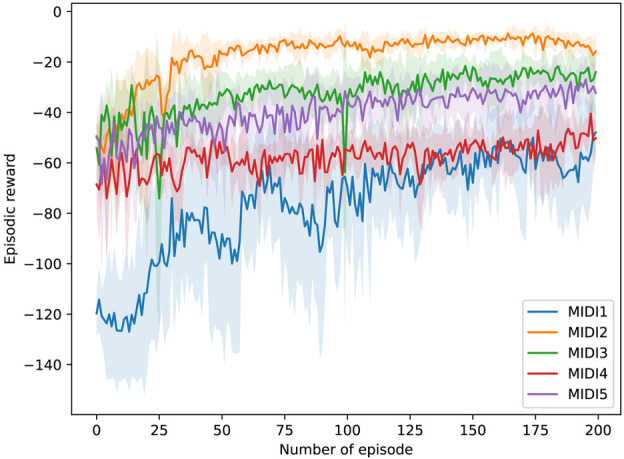
Training results in the real world setup using only extrinsic rewards. Five different MIDI inputs were used for the training experiments. For each input, the training is repeated ten times.

The training results show satisfying patterns. We repeated the training experiment for each MIDI input 10 times to have an average performance. In Figure 12, the average episodic rewards during training for MIDI inputs using only extrinsic reward. In Figure 12, the average episodic rewards during training for MIDI inputs using the combination of intrinsic and extrinsic reward. The intrinsic reward for each MIDI input is also shown separately. In each training experiment, the number of episodes (N) was 200, and the number of steps for each episode (T) was 25. With limiting the length of the MIDI inputs and the range of exploration in the latent space, the training lasts around 90 min each time. The critical point in the training data is that the episodic reward increases over time, which means that the algorithm can achieve the objectives defined in the experiment. However, we know that the optimal answer is not always in the range of the explored space.

**FIGURE 13:**
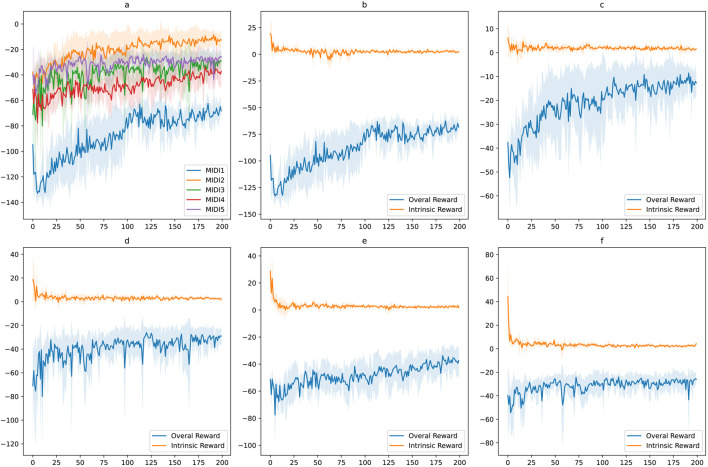
Training results in the real world setup using intrinsic and extrinsic rewards. **(A)** Comparing the average episodic rewards for the five MIDI inputs. **(B–F)** Average intrinsic rewards and overall rewards for each MIDI input.

**FIGURE 14:**
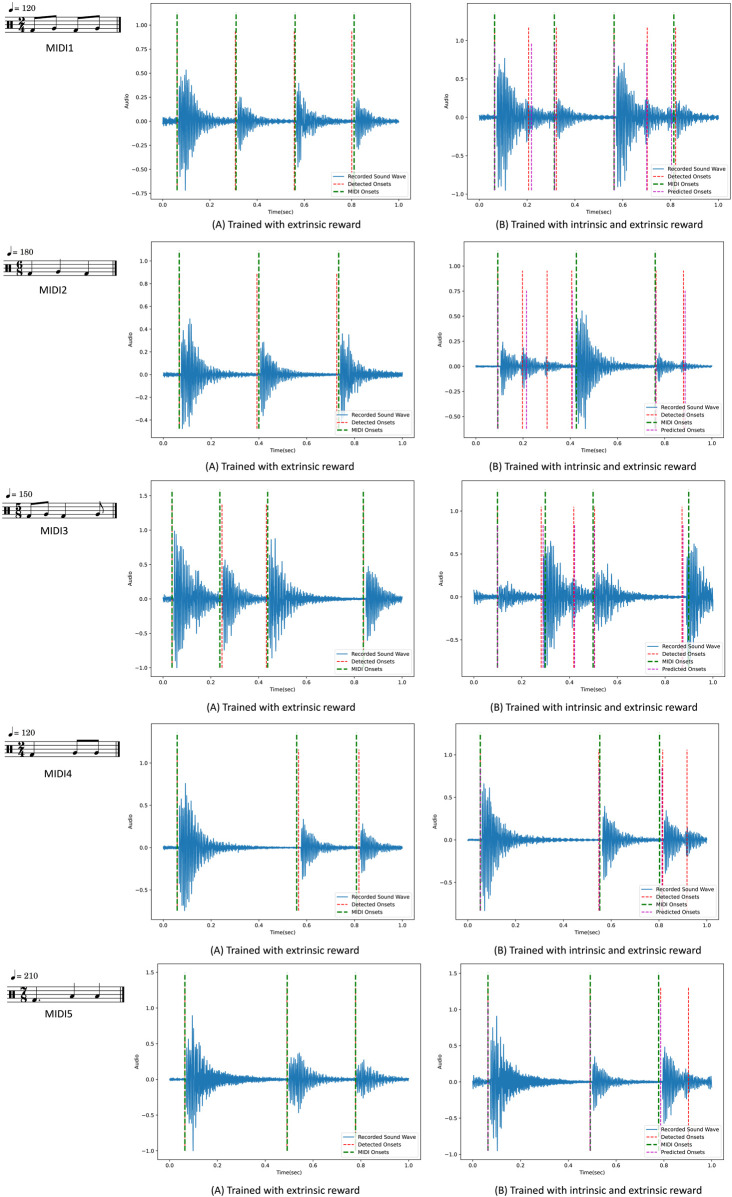
Comparing the drumming results after training with and without intrinsic rewards for each MIDI input. On the left side, the green lines indicate the input onsets set by the MIDI, and the red lines indicate the detected onsets from the audio. On the right side, the purple lines indicate the predicted onsets estimated by the internal model. The MIDI inputs are represented by musical notation here.

In Figures 13, 14, examples of the drumming results of the robots are shown after training. On the left side, the robots are only trained using the extrinsic reward, and on the right side, intrinsic reward was added. The predicted onsets are also depicted in the figures on the right side. Interestingly, as expected, the robots learn to play the MIDI inputs perfectly with only the extrinsic reward. However, adding the intrinsic reward reinforces the robots to look for emergent behaviour. In these examples, it can be seen that the reference notes given in the MIDI input are played, and at the same time, extra notes are also played, resulting from double and triple strokes. In other words, the robots are reinforced to use the rebounding effect of the flexible gripper to play the given rhythmic pattern. This behaviour is not just a result of variation in the training but is a natural output of exploring novel and meaningful behaviour. This behaviour is directly the result of the physical properties of the robots’ bodies.

As demonstrated in Figures 13, 14, we can see similar behaviour repeated for different inputs. Based on the results of the repeated training experiments, we can see that the robots can meet the objectives of the learning problem: 1) by using the extrinsic reward the robots perform the input patterns accurately, 2) by adding the intrinsic reward, robots can discover possible variations such as double strokes while following the MIDI onsets to keep the pattern accurate. The results can be evaluated from different perspectives. Objectively, we see that the accuracy of the performance is high using the extrinsic reward, and different variations are achieved using the intrinsic reward. However, for more subjective evaluation and studying the artistic value of the observed diversity, further research is required. The results can guarantee that the potential variations in the performance as a result of intrinsically motivated exploration can be exploited for artistic creativity.

## 5 Discussion

We find that combining intrinsic and extrinsic rewards can lead to creative behaviour in musical robots. Creativity is not the easiest quality to evaluate in robotics; however, comparing the robots’ behaviour using different training approaches can give some insights into possible creative results. While the subjective evaluation of creativity can be studied separately in another experiment, we try to focus on the process of learning and interpret the results objectively.

Training the robots using only extrinsic reward defined based on a reference MIDI file makes the results identical to the reference. This outcome is expected since the agent receives the highest reward when the drumming pattern is the closest to the given MIDI file. This approach is suitable when the robots are supposed to play a musical piece that is written in prior. Refining the reward function can help to optimize the performance of the robots. For instance, changing the weights on the timing or strength of the onsets can change the musical accent of the rhythmic pattern.

On the other hand, adding an intrinsic reward signal forces the robots to explore and search for novel and meaningful behaviour. The drumming patterns have rhythmic diversity when intrinsic reward is used during training. Looking for novelty is not a complex objective in RL. Adding a noisy signal to the agent’s actions can result in unseen observations. However, producing a random signal is not particularly creative; a novel pattern needs to make sense. A meaningful observation is usually compatible with previous observations. Using a prediction model enables the agent to measure whether a novel observation makes sense. When the novelty improves the prediction model, the observation is meaningful. Using the intrinsic reward based on this concept allows the training process to result in creativity. Especially in a musical context, prediction models play an essential role in human perception. The theory of predictive coding explains how internal models are fundamental in music perception (Koelsch et al., 2019). The drumming results of ZRob, when trained with intrinsic reward and the internal model, suggest possible creative musical expression.

The other aspect of our study is the role of the robots’ bodies in the training results. We know that multiple strokes drumming is directly affected by the robots’ flexible joints and their spring’s stiffness (Karbasi et al., 2022; Karbasi et al., 2023). During training with intrinsic reward, the robots find novel observations when the actions lead to multiple strokes. If the internal model learns to predict these novelties, the model can partly understand the dynamics of the robots’ bodies. In this way, the algorithm uses the physical properties of the robots to achieve meaningful behaviour. This corresponds to the embodied aspect of our robotic system. The robots’ computational part shapes the results we observe, but their body contributes significantly, as explained in the embodied intelligence framework.

In summary, we have found that 1) using extrinsic or intrinsic rewards for training drumming robots can result in optimised or creative musical performance and 2) how the embodied aspect of robotic drumming shapes the learning results of the system. Implementing intrinsically motivated reinforcement learning for drumming robots is the main contribution of our study, which has not been present in previous research in musical robotics.

### 5.1 Limitations and future works

A significant limitation for training robots in the real world is the hardware and computational resources. As mentioned previously, not all possible actions are feasible in real life. In addition, RL algorithms require many samples for training, which lasts for many hours. Even for a short MIDI file, it takes a long time for the algorithm to gather enough samples for training. Improving the RL algorithm can accelerate the training process and make it possible to design more complex tasks. In addition, other sound analysis methods can suggest richer musical analysis and make it possible to define different musical tasks for the robots. We only used onset detection methods since this study’s objective was rhythm. Using more sophisticated methods for analysing sound in real-time also requires high computational resources, making the system harder to train.

The performance results of the current study can be the topic of qualitative research on perceived creativity in robots. Also using the proposed approach in live performances or in an interactive setup with other human or robotic musicians can be studied in future works. The aim of this paper is a technical presentation of the system and subjective evaluation and applications of our approach can be the topic of further research.

## 6 Conclusion

In this study, we implemented an RL-based algorithm for training drumming robots called ZRob. We used DDPG architecture and intrinsic and extrinsic reward functions for training the robots. The training results suggest how extrinsic and intrinsic rewards can be applied to creativity and drumming performance. The developed system is an effort towards exploring embodied intelligence for musical performance. In future, the system can be improved by integrating pre-trained supervised models to make the training faster. In addition, using generative models that make MIDI files expands the capabilities of the robots to play AI-generated music in real-time.

## Data Availability

The datasets presented in this study can be found in online repositories. The names of the repository/repositories and accession number(s) can be found below: https://osf.io/zahfp/.
